# Lockdown Policies, Economic Support, and Mental Health: Evidence From the COVID-19 Pandemic in United States

**DOI:** 10.3389/fpubh.2022.857444

**Published:** 2022-06-02

**Authors:** Haitang Yao, Jiayang Wang, Wei Liu

**Affiliations:** Business School, Qingdao University, Qingdao, China

**Keywords:** COVID-19, lockdown policies, mental health, anxiety, depression, economic support

## Abstract

During the COVID-19 pandemic, various lockdown policies were put in place by the governments in different countries and different levels, which effectively curbed the spread of the virus, but also cause substantial damage to the mental health of local residents. We use statistics provided by the Household Pulse Survey and OxCGRT between 23 April 2020 and 30 August 2021 to analyze the impact of lockdown on overall mental health levels in US states during the COVID-19 pandemic at the macro level. The results show that the lockdown policies implemented by the state governments lead to a deterioration in psychological conditions, and this relationship varies to some extent depending on the level of high-quality economic support, that the state governments implement to alleviate the symptoms of depression and anxiety associated with the lockdown. Therefore, we argue that although lockdown policies are necessary during the COVID-19 pandemic, further government efforts are needed to give high-quality economic and mental health support to mitigate the negative effects of lockdown on mental health.

## Introduction

As of mid-December 2021, more than 27 million people worldwide have been diagnosed with pneumonia caused by COVID-19, resulting in more than 5.37 million deaths (1). In most countries severely affected by the pandemic, governments have taken measures to impose lockdowns and restrictions on outings to deter the uncontrolled spread of the virus, limit infections and deaths, and reduce the pressure on healthcare systems and healthcare providers. In the United States, most states and counties began implementing the lockdown policies in late March 2020, which were adopted by the local governments to reduce the spread of the virus under the pressure of unpredictable uncertainties posed by the pandemic (2).

In this study, what we concern about are the lockdown policies including stay-at-home orders, quarantine, social distancing, and isolation of confirmed patients associated with high risk. Studies have shown that government administration and public cooperation do lead to better control of pandemics like COVID-19 (3, 4). However, such lockdowns directly lead to reduction in social interaction and have negative impacts on individuals' mental health (5). For example, not only do people experience increased anxiety and stress about future income and employment (6), but they also face an immediate fear of infection, for themselves, their family or friends (7). Factors such as the incubation period of a pandemic and the time required for isolation may also lead to anxious emotional reactions (8). Restrictions on activities, loss of daily life and reduced social activities also result in feelings of boredom, depression, and isolation (9).

Numerous studies have shown that the COVID-19 pandemic caused severe psychological problems in the population (10–12), and we focused our research on the impact of lockdown policies on mental health. Existing research has explored the psychological effects of lockdown at the individual level and demonstrated that the mental health of individuals is influenced by a number of factors, such as work status, income, gender, and relationship status (13), as well as the length of lockdown, how and where they are imposed (14). Scholars have also found that lockdown affects different individuals in different ways and to different degrees. Many of them have compared the level of various mental health indicators measured in cross-sectional surveys conducted in the general population (15), Child (16), adolescents (17), adults (18), older adults (19), new mum (20), university students (21), and college students (22). For example, Yildirim (19) identified the psychosocial status, attitudes, and experiences of individuals aged 65 and older confined in their homes during the COVID-19 outbreak in Turkey, and concluded that lockdown applied specifically to older adults forced them to establish new routines and made them aware of some values; however, they asserted that they were stigmatized and isolated, their fear of COVID-19 increased, and they were treated unfairly. Olson et al. (22) conducted a photographic survey of college students' experiences during lockdown and found that students frequently reported deterioration in mental health. Non-academic aspects of students' lives, such as work and home environments, contributed significantly to perceived stress.

However, from a higher perspective, the lockdown policies developed by policymakers must have had a significant impact on individuals' mental health, and we argue that the interregional heterogeneity of such policies is an important but currently overlooked key factor. In this study, we discuss the impact of lockdown policies on overall American mental health at the state level, to provide policy recommendations for state governments to balance lockdown policies and individuals' mental health during the COVID-19 pandemic. Stress process theory suggests that adequate resources (e.g., high-quality social support) can prevent or mitigate the effects of stress on mental health (23–25). In particular, economic support as a positive intervention can reduce the impact of negative events on individuals, and therefore we consider the possible impact of government economic support policies on mental health.

We investigate such impact based on the statistical analyses provided by the Household Pulse Survey and the Oxford Covid-19 Government Response Tracker (OxCGRT) from 23 April 2020 to 30 August 2021. To our knowledge, this is probably one of the first study to track the mental health-related effects of state government lockdown policies at the macro level. Compared to other short-term studies at the individual level, our study better conveys the true effects of the “lockdown,” with the aim to provide a theoretical consideration for improving the adverse effects of lockdown policies on mental health and to contribute practical implications for improving individuals' mental health during the lockdown period.

## Methods

### Sample and Data Sources

We used the National Center for Health Statistics (NCHS) and the Census Bureau's ongoing Household Pulse Survey as our primary data sources. To detect changes of individuals' mental health during the COVID-19 pandemic, the NCHS, in collaboration with the Census Bureau, set up this survey dataset, which includes individual-level information on age, gender, race and ethnicity, educational attainment, and location, administered electronically to adults aged 18 years and elder in each U.S. state through an Internet-based questionnaire supplemented by email and text messaging. The survey began on 23 April 2020 and continues to date. Based on the survey data, we collected data from April 23, 2020 to August 30, 2021 and created panel data with a 12-day statistical period with a total sample of 1,734. In addition, we added data on government lockdown policies adopted during the COVID-19 pandemic from the OxCGRT. Our state-level control variables, such as the number of hospital beds, was obtained from the Statista database; the average hourly wage was from the Federal Reserve Economic Database (FRED); GDP and unemployment rates were from the US Bureau of Labor Statistics; all other control variables were from the OxCGRT.

### Measures

#### Dependent Variable

The dependent variable in this study is the degree of individuals' mental health. We used the estimates of depression and anxiety disorders published by the NCHS and the Census Bureau as proxies for measuring mental health, which has been shown to be an important measurement in previous studies (26–28). Higher estimates of depression and anxiety represent more severe mental health conditions.

#### Independent Variables

The independent variable is the government lockdown policies during the COVID-19 pandemic. We carefully selected the stringency index developed by the OxCGRT to measure the intensity of lockdown policies implemented by each state government (29). Specifically, the stringency index records the stringency of lockdown policies that restrict individuals' behavior, and is a composite measure consisting of eight restrictive indicators: school closures, workplace closures, cancellation of public events, restrictions on gatherings, public transport closures, stay-at-home requirements, restrictions on internal movement, and international travel controls.

#### Moderating Variables

In this study, the economic support policies adopted by the government during the COVID-19 pandemic are used as a moderating variable. This variable is also derived from the OxCGRT, from which we selected the economic support index to measure the intensity of economic support policies implemented by the government in each state (29). The index records the governments' economic policies and is also a composite measure that includes four indicators: income support, debt relief, fiscal measures, and international support.

#### Control Variables

We controlled for a number of regional pandemic and macroeconomic factors that may affect the estimates. We first controlled for the severity of the COVID-19 pandemic, measured as the number of confirmed cases in each U.S. state (30). The amount of available health care resources in each state may have an impact on individuals' mental health, and inadequate resources may cause panic and anxiety, thus we controlled for health care resources, measured as the natural logarithm value of the number of total hospital beds in each state. We then controlled for the intensity of vaccine policy implementation in each state, assigning values from small to large based on the range of people covered by vaccination (29). In addition, we controlled for some macroeconomic factors, including state GDP, average hourly wages, and unemployment rates for each state. Finally, we added regional dummies to control for unobserved heterogeneity across states.

### Estimation Models

We mainly considered two dependent variables, depression and anxiety disorders, in this study, and therefore two regression equations were included in our estimation models. The first equation examines the effect of government lockdown policies on individuals' depression and the moderating role of government economic support. The second equation examines the effect on individuals' anxiety and the moderating role of government economic support. In summary, the following OLS models were developed for the baseline regressions:


$$\begin{array}{l} Depression\;=\;\alpha_{0}\;+\;\alpha_{1}Lockdown_{it}\;+\alpha_{2}\;Lockdown_{it}\times Economic\\ \;Support_{it}+\sum Control_{it}\;+\delta_{t}+\mu_{it} \end{array}$$

where *i* = state, *t*= year, μ_*it*_ is the standard error term, and δ_*t*_ is the regional fixed effect.


$$\begin{array}{l} Anxiety\;=\;\beta_{0}+\beta_{1}\;Lockdown_{it}+\beta_{2}\;Lockdown_{it}\;\times Economic\\ \;Support_{it}\;+\sum Control_{it}\;+\gamma_{t}\;+\varepsilon_{it} \end{array}$$

where *i*= state, *t*= year, ε_*it*_ is the standard error term, and γ_*t*_ is the regional fixed effect.

## Results

### Descriptive Statistics and Correlation Results

Panel A of Table 1 shows the descriptive statistics and correlation results for all variables, except for the region dummies. To ensure that multicollinearity did not affect the estimation results, we calculated the variance inflation factors (VIFs), which are indicators of covariance between predictors. The results show that the VIFs for all variables are below 5.21 (mean = 2.49), well below the generally accepted threshold of 10.0 (31). We also tested the correlation coefficients between the variables, with a maximum value of 0.598, which is less than the acceptable value of 0.700 (32). Therefore, multicollinearity is not an important concern in our study.

Table 1Estimation results.

**Table:** 

**(A) Descriptive statistics and correlations**									
**Variables**	**Mean**	**S.D**.	**(1)**	**(2)**	**(3)**	**(4)**	**(5)**	**(6)**	**(7)**
(1) Depression	24.883	4.422	1.000						
(2) Lockdown	46.054	15.276	0.106	1.000					
(3) Beds	9.479	1.024	0.239	−0.084	1.000				
(4) Confirmed cases	11.319	1.884	0.133	−0.507	0.598	1.000			
(5) Earnings	28.154	3.224	−0.184	0.296	−0.037	0.120	1.000		
(6) Unemployment	7.693	3.441	0.028	0.500	0.124	−0.364	0.204	1.000	
(7) Vaccination	1.446	2	−0.333	−0.550	−0.006	0.571	0.056	−0.336	1.000
(8) Economic support	41.814	23.393	−0.085	0.316	−0.075	−0.152	0.352	0.195	−0.089

**Table:** 

**(B) OLS regression results of the relationship between lockdown and mental health**						
	**Model 1**	**Model 2**	**Model 3**	**Model 4**	**Model 5**	**Model 6**
**Variables**	**Depression**	**Depression**	**Depression**	**Anxiety**	**Anxiety**	**Anxiety**
Beds	0.058	0.074	0.192	−1.096^**^	−1.061^**^	−0.902^*^
	(0.374)	(0.381)	(0.386)	(0.455)	(0.460)	(0.464)
Confirmed cases	1.529^***^	1.627^***^	1.602^***^	2.043^***^	2.107^***^	2.074^***^
	(0.120)	(0.125)	(0.125)	(0.133)	(0.135)	(0.135)
Earnings	0.156	−0.084	−0.078	0.040	−0.151	−0.143
	(0.184)	(0.191)	(0.191)	(0.196)	(0.205)	(0.206)
Unemployment	−0.102^**^	−0.135^***^	−0.135^***^	−0.169^***^	−0.199^***^	−0.199^***^
	(0.044)	(0.045)	(0.045)	(0.046)	(0.047)	(0.047)
Vaccination	−1.614^***^	−1.498^***^	−1.478^***^	−2.287^***^	−2.185^***^	−2.158^***^
	(0.064)	(0.070)	(0.070)	(0.068)	(0.075)	(0.076)
Economic support	0.003	0.000	0.014	0.005	0.002	0.020^**^
	(0.006)	(0.006)	(0.009)	(0.007)	(0.007)	(0.009)
Lockdown		0.041^***^	0.043^***^		0.034^***^	0.036^***^
		(0.009)	(0.009)		(0.010)	(0.010)
Lockdown ×			−0.007^**^			−0.010^**^
Economic support			(0.004)			(0.004)
Constant	7.065	10.837^*^	9.344	21.129^***^	24.05^***^	22.04^***^
	(6.221)	(6.258)	(6.351)	(6.786)	(6.852)	(6.89)
Observations	1309	1305	1305	1309	1305	1305
R-squared	0.565	0.572	0.574	0.602	0.608	0.610
State dummies	Yes	Yes	Yes	Yes	Yes	Yes

* *p < 0.1;*

** *p < 0.05;*

*** *p < 0.01; Standard errors are in parentheses*.

### Empirical Tests

**Figure 1:**
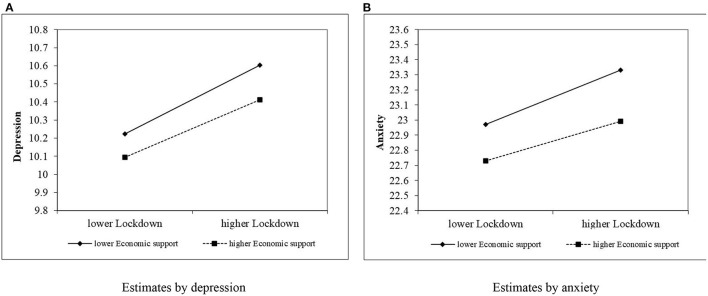
Moderating effect of economic support on the relationship between lockdown and mental health. **(A)** Estimates by depression, **(B)** Estimates by anxiety.

Panel B of Table 1 presents the OLS estimation results of the government lockdown policies and individuals' mental health. Models 1–3 test the impact of government lockdown policies on depression. Model 1 is a baseline model only including control and moderating variables. In Model 2, we added the independent variable, government lockdown policies (*Lockdown*), and the results suggest a significantly positive relationship between government lockdown policies and depression (α_1_ = 0.041, *p* < 0.01), in line with our expectation. We argued that the government economic support can mitigate the depression brought about by the lockdown, representing a negative moderating role. As shown in Model 3, the coefficient on the interaction term between government lockdown policies and government economic support (*Lockdown* × *Economic support*) is negative and significant (α_2_ = −0.007, *p* < 0.05), which supports our expectation. To gain more insight into this interaction, we plotted these relationships in Figure 1A (33). The figure shows that the positive relationship between the lockdown and depression is weaker as the intensity of government economic support is high, and stronger as the intensity of government economic support is low.

Models 4–6 examine the impact of government lockdown policies on anxiety disorders. Model 4 is a baseline model only including control and moderating variables. Similarly, we added the independent variable (*Lockdown*) in model 5 to examine the relationship between government lockdown policies and anxiety disorders. The results show a significantly positive relationship (β_1_ = 0.034, *p* < 0.01), in line with our expectation that lockdowns will lead to more severe mental health problems. Model 6 examines the moderating effect of government economic support, and the results show that the coefficient on the interaction term (*Lockdown* × *Economic support*) is negative and significant (β_2_ = −0.010, *p* < 0.05), suggesting that the relationship between the lockdown and anxiety disorders is weakened by higher levels of government economic support. Similarly, we plotted the slope of the simple regression reflected in Model 6. Figure 1B depicts the slope of the relationship. As can be seen, the slope of the line associated with lower economic support is significantly higher than the line associated with higher economic support.

## Discussion

The aim of this study is to examine the impact of the intensity of the lockdown policies imposed by the state governments on the mental health of U.S. individuals during the COVID-19 pandemic. We investigated our analyses based on the following two questions: (1) do government lockdown policies lead to a worsening of individuals' mental health? (2) to what extent does the relationship between lockdown policies and mental health vary across states depending on the level of government economic support? Specifically, we find that government lockdown policies are associated with increases in post-lockdown estimates of depression and anxiety disorders across states. Depending on the spread and infection of COVID-19, state governments have implemented different levels of lockdown policies. The most common types of lockdown are those requiring self-isolation and home quarantine of patients and close contacts, even urban closures in areas with severe outbreaks. Lockdowns appear to be the most effective way to deteriorate the spread of the virus, but it can lead to very serious mental health problems. It is well-documented that lockdowns trigger anxiety and insecurity (34, 35) and alter sleeping habits. During the lockdown, people sleep later than usual, stay in bed longer and sleep poorly (36). At the same time, anxiety and insecurity may be exacerbated by concerns about economic stress and major changes in daily life such as social isolation, possible viral infections, loss of loved ones, and home-schooling children (37). In addition to these phenomena, lockdowns can create social isolation and that people usually feel lonely after severe social isolation, and it is obviously that both loneliness and social isolation negatively affect mental health (38). Furthermore, we found that younger individuals in our sample were more likely to feel depressed and anxious than older ones, with emerging adults (aged 18–29) being the group most sensitive to the mental health impact of the COVID-19 pandemic. Emerging adulthood is a developmental period characterized not only by positive role transitions into full autonomy (e.g., living independently, entering the labor market, getting married), but also by high-risk behaviors such as heavy episodic drinking (39).

Secondly, our study also shows that economic support from the government alleviates the symptoms of depression and anxiety associated with the lockdowns. Another important source of stress is economic hardship (40), and economic support as a positive intervention can mitigate the impact of negative events on individuals. Lockdown brings social isolation and anxiety, where any unexpected event such as illness or accident becomes a psychological threat and burden to individuals, while a wealthy economic base will greatly increase the individuals' ability to resist physical and psychological risk. In addition, the economic downturn caused by the COVID-19 pandemic leads to a period of economic instability during which people face unemployment and low income and develop negative perceptions about their future lives, which lead to anxiety and depression. It has been shown that a reduction in income is the greatest predictor of the development of psychological disorders during the recovery period after the SARS outbreak (41). Therefore, high-quality economic support from the government during the pandemic may enable individuals to escape from their psychological conflict-induced anxiety state, to better adapt to their environment and cope with stress, and to increase resilience.

## Contributions and Practical Implications

This study makes several important contributions to the relationship between government lockdown policies and mental health during the COVID-19 pandemic. First, our study uses the data from the Household Pulse Survey and OxCGRT, takes the mental health values of all 50 states in the United States as the research samples, to examine the actual impact of government lockdown policies on mental health at the macro level, whereas most of the existing research on the relationship between lockdowns and mental health has been conducted at the individual level using first-hand survey data (7, 42, 43). Second, we found that most of the literature related to the pandemic lockdown to date has been dominated by short-term studies (8, 44), with a statistical time span of about 1 month, which does not allow for long-term tracking of the impact of lockdown policies implemented after the pandemic on mental health. Since our study covered nearly 2 years after the pandemic, we examined the impact of the government lockdown from 23 April 2020 to 30 August 2021, which better conveys the true effects of the lockdown than previous studies.

Furthermore, by exploring the relationship between the lockdown and mental health during the COVID-19 pandemic and the moderating effect of economic support, we aimed to provide a theoretical lens for ameliorating the negative impact of the lockdown on mental health and to provide practical strategies for improving the mental health of the population during the lockdown. On the one hand, from the government perspective, the implementation of lockdowns is necessary to deteriorate the spread of the pandemic (45). Although it can be effective in reducing the speed and extent of the pandemic, our study shows that it can be a significant threat to individuals' mental health. We have also demonstrated that government economic support can alleviate anxiety and depression, and we therefore suggest that the government should provide appropriate policy care for the isolated, such as income and debt support, unemployment subsidies for residents, and accelerate the establishment and improvement of an economic support system for the isolated to give them the courage to face the pandemic. Only with these measures can they face their study, work and life during the pandemic. On the other hand, from the individual's point of view, the quarantined can gain moral support and material care by confiding in or seeking help from colleagues, relatives and friends, thus enhancing their confidence in facing the tremendous pressure brought by the pandemic and relieving negative emotions and psychological stress.

## Limitations and Future Research Directions

There are several additional limitations to this study that need to be addressed. The first limitation relates to our measurement of regional mental health in the lockdown situation. We used only two measures of mental health, anxiety, and depression, in this study. Although these two measures are probably the most important indicators of mental health, subsequent studies will need to design and use tailored measures for psychosocial characteristics of different populations, such as loneliness and sleeping quality. In addition, due to the limitations of the dataset we used, we were unable to control for individual-level characteristics, which may have led to some bias in our results. Further research is expected to measure and compare in depth the effects of variables such as age, gender, education, work, and health conditions on mental health, providing more detailed and accurate information.

## Data Availability Statement

The original contributions presented in the study are included in the article/Supplementary Material, further inquiries can be directed to the corresponding authors.

## Author Contributions

All authors listed have made a substantial, direct, and intellectual contribution to the work and approved it for publication.

## Funding

This work was supported by the National Social Science Fund Youth Project of China (Grant No. 17CJY019).

## Conflict of Interest

The authors declare that the research was conducted in the absence of any commercial or financial relationships that could be construed as a potential conflict of interest.

## Publisher's Note

All claims expressed in this article are solely those of the authors and do not necessarily represent those of their affiliated organizations, or those of the publisher, the editors and the reviewers. Any product that may be evaluated in this article, or claim that may be made by its manufacturer, is not guaranteed or endorsed by the publisher.
