# Supplementary material for: Lockdown Policies, Economic Support, and Mental Health: Evidence From the COVID-19 Pandemic in United States
Source: Front Public Health. 2022 Jun 2;10:857444. doi: 10.3389/fpubh.2022.857444 (PMC9201054; doi:10.3389/fpubh.2022.857444)
Supplement: Supplementary file 1 [file Table_1.DOCX]

Supplementary Material

Referring to the mental health questionnaire survey conducted by NCHS in the United States, we conducted a similar survey in China using the same questionnaire items. In order to compensate for the missing part of the NCHS survey data on personal characteristics and respondents under 18 years old, we counted the characteristics of respondents such as gender, age, education level, marital status, income status, whether they were front-line workers of the pandemic and whether they were confirmed/suspected persons in the questionnaire, and extended the age range of respondents to those under 18 years old. For the lockdown policy section, we investigated the strictness of China’s implementation of the lockdown policy by referring to OxCGRT’s measurement items that investigated six aspects of school closures, workplace closures , restrictions on gatherings, public transport closures, stay-at-home requirements, and international travel controls, and thus used similar variable measures and methods to conduct the original data survey, which resulted in 1,642 valid questionnaires. The regression results are shown in Supplementary Table 1, from which we can see that the results are still consistent with the original results after conducting a similar survey.

**Supplementary Table 1.** Estimation results

| **Panel A: Descriptive statistics and correlations** | | | | | | | | | | | | | | | |
| --- | --- | --- | --- | --- | --- | --- | --- | --- | --- | --- | --- | --- | --- | --- | --- |
| **Variables** | **Mean** | **S.D.** | | **(1)** | **(2)** | | **(3)** | **(4)** | **(5)** | | **(6)** | **(7)** | | **(8)** | **(9)** |
| (1) Depression | 2.103 | 0.944 | | 1.000 |  | |  |  |  | |  |  | |  |  |
| (2) Lockdown | 4.99 | 1.667 | | 0.060 | 1.000 | |  |  |  | |  |  | |  |  |
| (3) Sex | 1.652 | 0.476 | | 0.037 | 0.032 | | 1.000 |  |  | |  |  | |  |  |
| (4) Age | 1.459 | 0.922 | | -0.187 | -0.030 | | -0.020 | 1.000 |  | |  |  | |  |  |
| (5) Edu | 5.245 | 0.873 | | 0.042 | -0.078 | | 0.015 | -0.135 | 1.000 | |  |  | |  |  |
| (6) Marriage | 1.783 | 0.441 | | 0.208 | 0.043 | | 0.031 | -0.627 | -0.010 | | 1.000 |  | |  |  |
| (7) Wige | 2 | 1.363 | | -0.168 | -0.079 | | -0.082 | 0.473 | 0.215 | | -0.512 | 1.000 | |  |  |
| (8) Worker | 1.924 | 0.265 | | 0.017 | -0.032 | | 0.031 | -0.049 | 0.002 | | 0.093 | -0.125 | | 1.000 |  |
| (9) Ifconfirm | 3.974 | 0.222 | | 0.037 | -0.033 | | 0.043 | -0.032 | 0.067 | | 0.037 | 0.020 | | -0.012 | 1.000 |
| **Panel B: OLS regression results of the relationship between lockdown and mental health** | | | | | | | | | | | | | | | |
|  | | | **Model 1** | | | **Model 2** | | | | **Model 3** | | | **Model 4** | | |
| **Variables** | | | **Depression** | | | **Depression** | | | | **Anxiety** | | | **Anxiety** | | |
| Sex | | | 0.045 | | | 0.042 | | | | 0.087^*^ | | | 0.084^*^ | | |
|  | | | (0.049) | | | (0.048) | | | | (0.050) | | | (0.050) | | |
| Age | | | -0.062 | | | -0.061^*^ | | | | -0.066^*^ | | | -0.066^*^ | | |
|  | | | (0.038) | | | (0.037) | | | | (0.038) | | | (0.038) | | |
| Edu | | | 0.056^**^ | | | 0.060^**^ | | | | 0.096^***^ | | | 0.100^***^ | | |
|  | | | (0.027) | | | (0.027) | | | | (0.028) | | | (0.028) | | |
| Marriage | | | 0.268^***^ | | | 0.267^***^ | | | | 0.159^**^ | | | 0.157^**^ | | |
|  | | | (0.075) | | | (0.074) | | | | (0.074) | | | (0.073) | | |
| Wige | | | -0.06^***^ | | | -0.057^***^ | | | | -0.047^**^ | | | -0.046^**^ | | |
|  | | | (0.022) | | | (0.022) | | | | (0.023) | | | (0.023) | | |
| Worker | | | -0.032 | | | -0.024 | | | | -0.079 | | | -0.072 | | |
|  | | | (0.089) | | | (0.088) | | | | (0.088) | | | (0.088) | | |
| Ifconfirm | | | 0.119 | | | 0.125 | | | | 0.208^**^ | | | 0.214^***^ | | |
|  | | | (0.109) | | | (0.109) | | | | (0.081) | | | (0.081) | | |
| Lockdown | | |  | | | 0.029^**^ | | | |  | | | 0.026^*^ | | |
|  | | |  | | | (0.014) | | | |  | | | (0.014) | | |
| Constant | | | 1.054** | | | 0.855 | | | | 0.662 | | | 0.480 | | |
|  | | | (0.517) | | | (0.524) | | | | (0.425) | | | (0.435) | | |
| Observations | | | 1642 | | | 1642 | | | | 1642 | | | 1642 | | |
| R-squared | | | 0.056 | | | 0.058 | | | | 0.041 | | | 0.043 | | |

Note: ^*^p < 0.1; ^**^p < 0.05; ^***^p < 0.01; Standard errors are in parentheses.
